# The Role of Lyophilized Xenodermotransplants in Repairing the Atria’s Structure and the Peculiarities of Regenerative Processes after Thermal Trauma in an Experiment

**DOI:** 10.3390/life13071470

**Published:** 2023-06-29

**Authors:** Adam Osowski, Iryna Hetmaniuk, Olena Fedchyshyn, Mykhailo Sas, Yuliia Lomakina, Nataliia Tkachuk, Olena Budarna, Volodymyr Fik, Larisa Fedoniuk, Joanna Wojtkiewicz

**Affiliations:** 1 Department of Human Physiology and Pathophysiology, University of Warmia and Mazury in Olsztyn, 2 Oczapowskiego Street, 10-719 Olsztyn, Poland; 2 Medical Biology Department, Horbachevsky Ternopil National Medical University, 2 Yu. Slovatskyi Street, 46001 Ternopil, Ukraine; getmanyuk@tdmu.edu.ua (I.H.); fedchyshyn_oliv@tdmu.edu.ua (O.F.); sas_mype@tdmu.edu.ua (M.S.); tkachuk@tdmu.edu.ua (N.T.); budarna@tdmu.edu.ua (O.B.); fedonyuklj@tdmu.edu.ua (L.F.); 3 Department of Medical Biology and Genetics, Bukovinian State Medical University, 15 Yu. Fedkovich Street, 58000 Chernivtsi, Ukraine; lomakinajulia@bsmu.edu.ua; 4 Department of Normal Anatomy, Danylo Halytsky Lviv National Medical University, 69 Pekarska Street, 79010 Lviv, Ukraine; fikvolodymyr@ukr.net

**Keywords:** atria and auricles of the heart, morphological changes, microscopy, microscopy, thermal trauma, biological lyophilized xenografts, tissue regeneration

## Abstract

The effects of severe burn injuries on the cardiovascular system, specifically the atria and auricles of the heart, were investigated. The potential benefits of using lyophilized xenodermotransplants as a treatment option were also evaluated. The experiments were conducted on adult guinea pigs divided into three groups: intact animals, animals with burns, and animals with burns who underwent early necrectomy followed by wound closure with lyophilized xenodermotransplants. Third-degree burns caused significant ultrastructural changes in atrial cardiomyocytes, leading to long-term destructive changes in the structural components of the atria. However, the use of lyophilized xenodermotransplants had a positive effect on the atrial ultrastructure over time. This study highlights the complex and varied effects of burn injuries on the body and the potential benefits of lyophilized xenodermotransplants in treating severe burn injuries. By preventing destructive changes in the heart and activating regenerative processes, lyophilized xenodermotransplants can improve the condition of the heart after thermal injury. Further research and development in this area are necessary for understanding the potential of lyophilized xenodermotransplants in tissue repair and regeneration.

## 1. Introduction

Burn injuries have been a significant issue in theoretical and practical medicine for many years. According to the World Health Organization (WHO), burns rank third among other traumatic injuries, and the number of victims in industrialized countries is steadily increasing. The urgency of the problem of thermal injuries is determined by their relatively high frequency in everyday life and production, the severity of burns, their complexity, and the duration of patient treatment, which results in frequent disability and high mortality rates [1,2,3,4]. Burn injuries also commonly occur during military actions due to the burning of military equipment and the use of incendiary mixtures [5,6].

It is well known that deep and large burns on the skin can cause significant structural and metabolic disorders in the body’s systems, organs, and tissues, including the heart [7]. These changes are often caused by the exogenous and endogenous intoxication of the body, with burn wounds being a frequent source. Therefore, the use of tools to reduce the level of toxins in the body is promising for the treatment of severe burns [8,9,10,11]. In recent years, lyophilized xenodermografts have increasingly been used in combustiology to temporarily close wound surfaces and repair biological membranes [12]. Applying Xeno-Skin to a wound that has been cleansed of dead cells prevents the progressive intoxication of the body from the wound, reduces the loss of water, proteins, and trace elements from the wound’s surface, and helps to restore the skin and to regenerate tissues in a shorter time. This, in turn, has a positive effect on morphofunctional health [13,14,15,16].

The search for the ideal skin substitute, which should be elastic, strong, and tightly adherent to the wound’s surface, as well as reduce pain, prevent scarring, and reduce the treatment time of patients, has led to the increased use of pig skin xenografts for temporarily closing burn wounds in recent years. Observations have shown that pig skin xenografts are easy to apply to wounds, fit tightly to the granulations, provide wound relief, retain drainage properties, and are easily removed during the next dressing [17]. The inclusion of pig skin transplantation in a complex of therapeutic measures in patients allows for an improvement of the condition of granulation tissues, an increase in patient survival, and reductions in the manifestations of burn toxemia, the duration of treatment, and the preoperative preparation time.

However, there is insufficient morphological research in the scientific literature on the effect of severe burns on the structural components of the heart while using xenodermotransplants. This study aimed to establish the patterns of morphological and morphometric changes in the atria and auricles of the heart after severe thermal injury while using lyophilized xenodermotransplants.

## 2. Materials and Methods

### 2.1. Study Design

The present study investigated the effect of lyophilized xenodermotransplants on the atria and auricles of the heart in guinea pigs with severe thermal injury. A total of 48 adult guinea pigs were divided into three groups: intact animals (6 animals), animals with burns (24 animals), and animals with burns that underwent early necrectomy followed by wound closure with lyophilized xenodermotransplants (18 animals).

The burn injuries were induced using a method developed at the Department of Bio-chemistry and Histology of Ternopil State Medical University. The animals were subjected to water vapor under general ether anesthesia at a temperature of 96–97 °C for 60 s. The water vapor was applied to the epilated surface of the back, resulting in a IIIA–IIIB-degree burn covering 18–20% of the body surface.

The damaged skin was then covered with lyophilized xenodermotransplants. Animals in the second group were decapitated on days 1, 7, 14, and 21, corresponding to the stages of early and late toxemia: shock, early and late toxemia, and septicotoxemia, respectively. Animals in the third group were decapitated on days 7, 14, and 21 of the experiment.

This study focused on the atria and auricles, which play an important role in the body’s contractile and secretory functions. Atrium and auricle are two structures of the upper portion of the heart. Atrium is a chamber that supplies blood to the ventricles. Auricle is an outer appendage of the atrium.

Overall, the results of this study could provide valuable insights into the effects of lyophilized xenodermotransplants on the structural components of the heart in guinea pigs with severe thermal injuries.

### 2.2. Light Microscoping Investigation

For the purpose of our microscopic analysis, samples of the middle portion of tissue from the atria and auricles were extracted, fixed in a 10% neutral formalin solution, dehydrated, and embedded in paraffin blocks. The resulting sections, obtained using a microtome, were stained using hematoxylin and eosin. The histological samples were observed using an SEO-SCAN light microscope and captured in photographs through the use of a Vision CCD Camera.

### 2.3. Morphometric Analyses

Morphometric and quantitative analyses were conducted using a histological specimen visual analysis system. Images were displayed on a computer monitor through a LOMO Biolam microscope connected to a Vision CCD camera and InterVideoWinDVR software. Morphometric studies were performed using VideoTest-5.0, CAARA Image Base, and Microsoft Excel software on a personal computer. The study was conducted at designated time intervals during the experiment using hematoxylin–eosin-stained specimens. The following parameters were evaluated: muscle-to-connective tissue ratio, relative volume of blood vessels, cardiomyocyte thickness, and nucleus size.

### 2.4. Electron Microscopy

For electron microscopy analysis, the atrial tissue samples were fixed in a 2.5% glutaraldehyde solution and then post-fixed in a 1% osmium tetraoxide solution in phosphate buffer. The samples were further processed using a standard protocol [18]. Ultrathin sections were obtained using an LKB-3 ultramicrotome and contrasted with uranyl acetate and lead citrate according to Reynolds’ method. The sections were examined under a PEM- 125 K electron microscope for analysis.

### 2.5. Statistical Analyses

The digital data obtained from the experiment were organized and processed using methods of variation statistics with the Student’s criterion.

## 3. Results

### 3.1. Microscopic Examination of the Atria and Auricles of the Heart on the 7th Day, the Stage of Late Toxemia of Burn Disease on the 14th Day and 21st Day of the Experiment

The microscopic examination of the atria and auricles of the heart on the 7th day of the experiment showed that, in response to the thermal damage in their walls, they developed compensatory and adaptive reactions as well as destructive changes. These manifested as changes in the bloodstream and extravascular space. In the myocardium, there was an increase in the relative volume of the connective tissue component, and there was a stratification of muscle fibers. In the atria, the relative volume of muscle fibers decreased by 1.09 times compared to that of the intact animals. The percentage of loose fibrous connective tissue and blood vessels increased, respectively, 1.36 and 1.25 times that of the normal range. The same violations of the relationship between the structural components of the myocardium were observed in the auricles of the heart (Table 1).

During the late stage of toxemia in burn disease (14th day), it was found that the atria and auricles of the heart exhibited destructive changes in response to thermal damage, as indicated by previous studies [19,20,21]. The myocardial vascular bed exhibited significant changes, including an increase in connective tissue edema and significant stratification of muscle fibers. The morphometric analysis revealed a decrease in the relative volume of the muscle fibers in the atria and auricles by 1.12 and 1.09 times, respectively, compared to that of the intact animals. This decrease was attributed to the replacement of muscle structures by an increase in the relative volume of swollen loose fibrous connective tissue, which increased 1.72 times in the atria and 1.46 times in the auricles compared to those of the intact animals. While the percentage of vessels in the atrial and auricular myocardium increased insignificantly relative to their normal percentages (see Table 1), there were vessels with closed lumens in addition to varicose and blood-filled veins.

On day 21 of the experiment, significant and irreversible damage occurred in the atria and auricles of the heart, resulting in profound changes in both the muscle and connective tissue components of the myocardium. In the atria, there was a 2.07-fold increase in the percentage of loose connective tissue compared to that of the intact animals, while the relative volume of muscle fibers decreased by 1.11 times compared to the normal range. Unlike in previous stages of the study, a decrease in the relative volume of blood vessels was observed due to the collapse of vessel lumens. Similar changes were observed in the auricles of the heart, with a decrease in the relative volume of muscle fibers and blood vessels and an increase in the percentage of loose connective tissue (refer to Table 1 for details).

Histological studies of the atria and auricles of the heart showed that early necrectomy followed by wound closure with lyophilized xenodermotransplants on the 7th day of the experiment provides a better preservation of the structural components of the myocardium and activates regenerative processes.

The results of the morphometric studies of the atrial myocardium showed that the relative volume of muscle fibers decreased 1.07 times from that of the intact animals, but increased 1.02 times from that of the animals with thermal injury. There was an increase in the percentage of loose connective tissue (1.25 times) and blood vessels (1.21 times), relative to the corresponding indicators of the intact animals. Compared to the animals of the second group, the relative volumes of these structural components of the myocardium were 1.09 and 1.04 times lower, respectively. In the auricles of the heart, the relationships between the structural components of the myocardium were less pronounced than those in the atria. The relative volume of muscle fibers increased 1.02 times relative to that of the burnt animals but was 1.04 times lower than normal. The proportional volume of connective tissue increased 1.18 times in comparison to the rate of the intact animals and did not differ significantly from the corresponding proportion in the burnt animals during this period (Table 2).

The microscopic studies performed on the 14th day of the experiment showed that the use of Xeno-Skin showed significantly a better preservation of the structural components of the myocardium of the atria and auricles of the heart compared with that of the animals without correction [18]. Morphometrically, it was found that the relative volume of connective tissue in the atria and auricles increased by 1.18 and 1.07 times, respectively, relative to the corresponding rates of the intact animals, but was 1.45 and 1.35 times lower, respectively, than those of the burnt animals. In the atria and auricles, there was an increase in the relative volume of muscle fibers compared to the previous period of the experiment and that of the animals of the second group. However, the relative volume of atrial and auricular myocardial muscle fibers was reduced by 1.05 and 1.03 times, respectively. The use of Xeno-Skin had a positive effect on the vascular bed of the myocardium. However, at this time of the experiment, the number of vessels with dilated and blood-filled lumens was smaller compared to that of the animals without correction. Moreover, their relative volume was higher than the corresponding indicators of the animals in the first and second groups (see Table 2). As we were concerned that the myocardium was not observed in the vessels, the lumens of these were connected.

The present study aimed to investigate the effect of using Xeno-Skin for wound closure on the structural components of the myocardium of the atria and auricles of burnt animals. A microscopic analysis performed on the 21st day of the experiment revealed that Xeno-Skin had a positive impact on the structural composition of the myocardium [19]. The morphometric data supported this finding and demonstrated a favorable effect of Xeno-Skin on the ratio of the structural components of the myocardium.

In the atria and auricles of the heart, the relative volume of muscle fibers increased by 1.09 and 1.1 times, respectively, compared to animals in the second group, but this increase was not statistically significant compared to intact animals. However, it is worth noting that the relative volumes of blood vessels and connective tissue in this period were comparable to those of the normal animals. Moreover, the percentage of loose connective tissue in the atria and auricles decreased significantly by 1.78 and 1.65 times, respectively, compared with that of the animals without correction, indicating a reduction in connective tissue edema and no expansion of perivascular spaces (see Table 2).

Taken together, these results suggest that the use of Xeno-Skin for wound closure can help preserve the structural components of the myocardium of the atria and auricles of burnt animals and mitigate the negative effects of burn injury on the heart.

### 3.2. Submicroscopic Examination of the Atria and Auricles of the Heart on the 7th Day, the Stage of Late Toxemia of Burn Disease on the 14th Day and 21st Day of the Experiment

**Figure 1:**
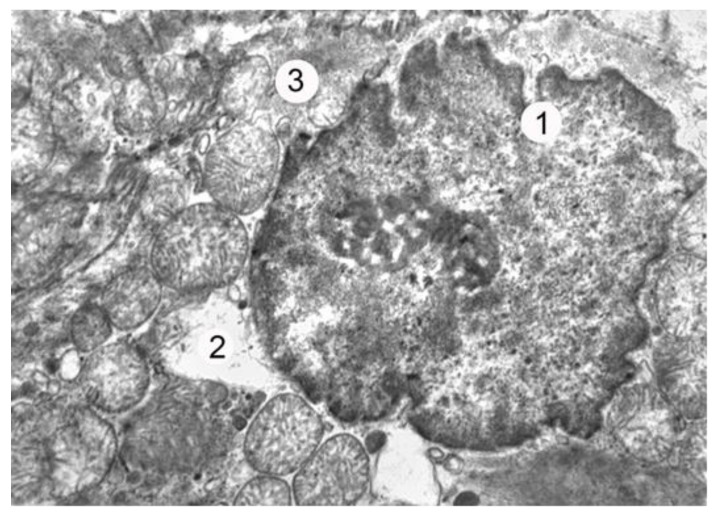
Fragment of a cardiomyocyte of the second group of animals of the left atrium one day after thermal injury. Deep karyolemma invaginations (1); mitochondria with enlightened matrix (2); degranulated cytoplasm (3) ×14,000.

On the first day of the experiment, which corresponded to the stage of burn shock, electron microscopic investigations revealed notable changes in the energy apparatus of the atrial myocytes. Specifically, the hypertrophy of most mitochondria was observed, along with a partial reduction in their cristae and an illuminated matrix. These findings collectively suggest an activation of energy metabolism in the cell. The local thickening and loosening of myofibrils, along with partially fragmented myofilaments, were also observed in the contractile apparatus of the cardiomyocytes, albeit at a relatively low prevalence during this period of the experiment. Additionally, the degranulation of most of the myoendocrine cells of the heart was detected during the burn shock phase, with only isolated granules found in certain myocytes, primarily concentrated near the sarcolemma. The relatively low concentration of specific secretory granules in the cytoplasm of atrial myocytes indicates the active excretion of atrial natriuretic peptide (ANP) from the cell into the bloodstream, as depicted in Figure 1.

On the 7th day of the experiment, a submicroscopic examination was conducted on the atrial cardiomyocytes to investigate the impact of the impaired blood supply to the heart on intracellular changes. The findings revealed a significant increase in intracellular changes which were reflected in the hypertrophy of most mitochondria, enlightenment of their matrix, and damage to the cristae. Notably, some of these organelles had destroyed areas of the outer mitochondrial membrane. Nonetheless, alongside the damaged organelles, mitochondria with moderate structural changes were also identified, with their outer and inner membranes retaining their integrity.

Furthermore, changes in the contractile apparatus of the cell were observed, including the thinning of myofibrils and their partial lysis, along with areas of overshortening with a disorderly arrangement of sarcomeres. Moreover, compared to the previous term, the current term showed an activation of synthetic processes in the cell. This activation was manifested by the dilation of endoplasmic reticulum tubules, the hypertrophy of the Golgi apparatus, and an increase in the amount of prohormones in the cell.

The accumulation of specific secretory granules in the perinuclear part of the cytoplasm of the atrial myoendocrine cells further indicated that the cell undergoes the processes of the synthesis and accumulation of secretions. Overall, the established submicroscopic changes in cardiomyocytes reflect the course of their adaptive and compensatory reactions, emphasizing the importance of investigating such cellular adaptations in the context of heart disease.

On the 21st day of the experiment, a significant increase in deleterious changes was observed, many of which were deemed irreversible. Most of the cardiomyocytes exhibited pyknotic nuclei with an electron-dense karyoplasm and a poorly expressed perinuclear space. Profound destructive changes in the mitochondria were apparent, as evidenced by hypertrophy, a disruption of the outer and inner membranes, the destruction of a majority of the cristae, and a clarification of their matrix. The contractile apparatus of the cell was markedly impaired, characterized by myofibril fragmentation and lysis, myofilament damage, and the disarrayed arrangement of sarcomeres. Atrial cardiomyocytes did not display any specific secretory granules in the cytoplasm. Concurrently, extensive destructive changes in the endoplasmic reticulum and Golgi apparatus were noted, characterized by the expansion, vacuolation, and fragmentation of their structural components. The absence of granules in the cytoplasm of the atrial myoendocrine cells and damage to the components of the synthetic apparatus in these cells indicated the cessation of atrial natriuretic peptide secretion and the loss of the heart’s ability to regulate the body’s water–electrolyte balance.

Contemporary scientific literature provides extensive coverage of the typical endocrine effects of atrial natriuretic peptides, which play a crucial role in regulating cardiovascular homeostasis by increasing natriuresis and diuresis, as well as inhibiting the renin–angio tensin–aldosterone system. In recent years, new data have emerged on the vital role of this hormone in regulating autocrine and paracrine functions in relation to the coronary circulation of the heart.

**Figure 2:**
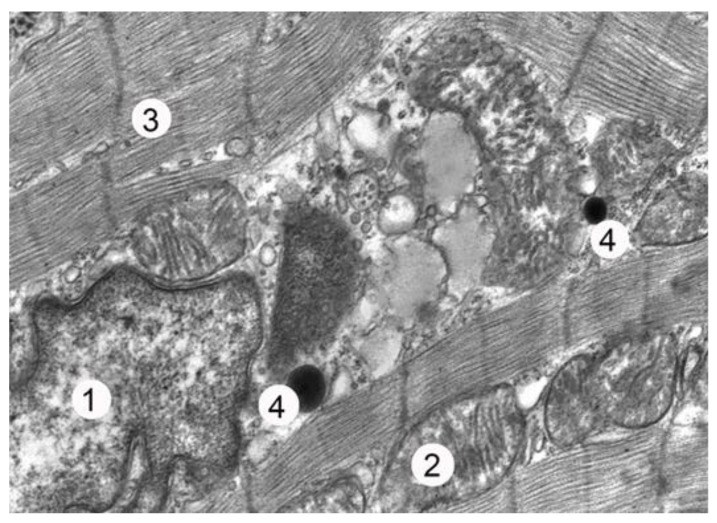
Fragment of atrial cardiomyocyte of the third group of animals on the 7th day after thermal injury under the conditions of lyophilized xenodermotransplants. Nucleus with karyolemma (1), mitochondria (2), myofibrils (3), and secretory granules (4) ×20,000.

The ultrastructural studies of atrial myocytes in animals from the third experimental group revealed that early necrectomy with subsequent wound closure using lyophilized xenodermotransplants on the 7th day of the experiment resulted in the better preservation of their structural components compared to that of the animals in the second group. In many cardiomyocytes, significant intussusception of the karyolemma was observed, along with enlarged nucleoli and karyoplasm dominated by euchromatin and ribosomal granules, indicating nuclear activation. The cytoplasmic changes were less pronounced compared to those of the animals in the second experimental group. The myofibrils had relatively well-preserved sarcomeres and myofilaments, with only some areas showing thinning and lysis. Partially destroyed cristae and an enlightened matrix were observed in some mitochondria. During this period, there was an expansion of the tubules of the endoplasmic reticulum and tanks of the Golgi apparatus, as well as an increase in vacuoles. The number of secretory granules in the cells was low, with small groups located in the perinuclear zone of the sarcoplasm, between single myofibrils, and near the sarcolemma (Figure 2).

An electron microscopy analysis of the atrial cardiomyocytes after 14 days of the experiment revealed a prevalence of regenerative processes over destructive–degenerative processes. Compared to the second group of animals, the cells showed less damage to their cytoplasmic and nuclear membranes. The nuclei were enlarged, euchromatin was predominant, and karyoplasm contained numerous ribosomal granules. A moderate amount of organelle destruction was observed in the sarcoplasm of atrial cardiomyocytes, with a higher number of ribosomes and polisomes and fewer autophagos. The myofibrils displayed moderate destruction but retained their clear sarcomeric structure. The mitochondria showed hypertrophy and hyperplasia, indicating heteromorphic changes. The tubules of the endoplasmic reticulum and the Golgi tank were moderately dilated. The cytoplasm contained an increased number of secretory granules, which were located near the nucleus, partly between myofibrils, and near the plasmalemma. These findings suggest an active synthesis of natriuretic hormones by myocytes.

The electron microscopy analysis conducted on the 21st day of the experiment revealed that the lyophilized xenodermotransplants effectively promoted the renewal of the intracellular structures in the heart. A majority of the cardiomyocytes showed no structural changes in theatrial nuclei, and no significant changes were observed in the contractile apparatus in the sarcoplasm of the cardiac muscle cells. The myofibrils maintained a normal structure, arranged in the order of the sarcomeres, with only a few areas showing thinning, defoliation, and partial lysis. The mitochondria exhibited heteromorphic changes, with most cells containing well-contoured cristae and outer membranes. However, some cells displayed partial crystal destruction and local matrix enlightenment. No significant changes were found in the structures of the endoplasmic reticulum and the Golgi apparatus, with moderate lumens in the tubules and cisterns of these organelles. A significant accumulation of specific secretory granules was found near the nucleus, as well as small groups of granules located between myofibrils and near the sarcolemma. These granules had different sizes and densities, indicating the restoration of the phase nature of the endocrine function of the cardiacmyocytes. During this period of the experiment, the normalization of intercellular space and contacts was established. The intercalated discs were contoured, had intact desmoso mal and slit junctions, and had a characteristic tortuous course.

## 4. Discussion

Since the 1970s, there has been a heightened interest in the use of xenografts as a substitute for human tissue. This has paved the way for new treatment modalities in addition to being used as a model to evaluate the safety and effectiveness of therapeutic trials. The xenograft is efficacious in the treatment of periodontal implants, cutaneous wounds, and gastro-intestinal cancer [20].

Skin wounds are a significant burden on the healthcare system with millions of people affected worldwide. Burn injuries have been a major concern in the field of theoretical and practical medicine for a long time. These injuries are frequently observed during military actions due to burns after using phosphorus weapons that contain white phosphorus, which release an incendiary compound with a burning temperature of over 800 °C [5]. Moreover, burns occur in children when rules for handling fire are not followed [6].

Modern medical technology is geared towards novel dressing materials [21,22]. Skin substitutes can be used in many wounds, including extensive burns, donor sites, residual wounds, skin cancer, peristomal pyoderma gangrenosum, diabetic foot ulcers, vasculopathic ulcers, venous status ulcers, radiation dermatitis, and surgical and traumatic wounds [23,24,25].

The significance of the problem of thermal injuries is attributed to their relatively high occurrence rate both at home and in the workplace. Burn disease can result from thermal trauma, which causes damage to all body systems capable of self-regulation. The severity of burn disease, the complexity, and the duration of patient treatment, along with their high disability and mortality rates, further compound this issue. When a thermal injury occurs, it results in the total or partial destruction of the skin and underlying tissues [26,27,28,29]. Burns not only destroy the skin directly under the influence of the thermal agent but also lead to ischemic processes in the tissues, which can cause further skin destruction. Deep and large burns on the skin can cause significant structural and metabolic disruptions in the body’s systems and organs, including the heart. Morphological and functional changes in the area of the burn wound are the triggering mechanism for pathological changes [30,31].

The remodeling of the myocardium during heart failure affects all components of cardiac muscle cells. It is especially apparent that the myofibrils and the sarcomere elements show early and significant changes, resulting in the disappearance of sarcomeres and the loss of contractile function in cardiac muscle cells. This phenomenon affects the myocytes heterogeneously, with focal changes of varying severity. Finally, the overall altered state of the cells is primarily caused by the loss of sarcomeres [32,33].

Apart from the lack of sarcomeres, the reduction in titin, which is the “third” sarcomeric filament system, is also responsible for the decreased compliance of failing hearts. A reduction in titin was found in the same myocytes that lacked contractile filaments [34]. Experimental studies in isolated adult myocytes in culture have shown that the expression and arrangement of titin filaments are the prerequisites for the formation of intact sarcomeres. While the ratio of unspecified cytoplasm to myofilaments increased, the cytoskeletal structures showed a significant augmentation combined with disorganization, which is most probably a compensatory mechanism for the loss of sarcomeres [35,36].

The left ventricle and atria of the heart have garnered significant attention from researchers due to the presence of specific secretory granules in their muscle cells. These granules were first described in a publication by Kisch over 50 years ago [37]. In 1964, S. Jamieson and G. Polade noted that atrial cardiomyocytes in mammals contain specific secretory granules, which are structurally similar to those found in some endocrine organs and respond to changes in the water and electrolyte balance in the organism. Research in this area gained momentum in 1981 when De Bold and co-authors [38] experimentally demonstrated that introducing atrial homogenate to animals resulted in increased natriuresis and diuresis. A hypothesis was proposed that the granules of atrial cardiomyocytes contain a specific substance capable of influencing the water–electrolyte balance of the organism, known as the atrial natriuretic peptide.

According to the majority of authors [39,40], the atrial natriuretic peptide is primarily synthesized and secreted by atrial cardiomyocytes in response to an increase in atrial pressure and volume. This leads to an increase in the excretion of sodium and water from the organism and a decrease in blood pressure.

**Scheme 1:**
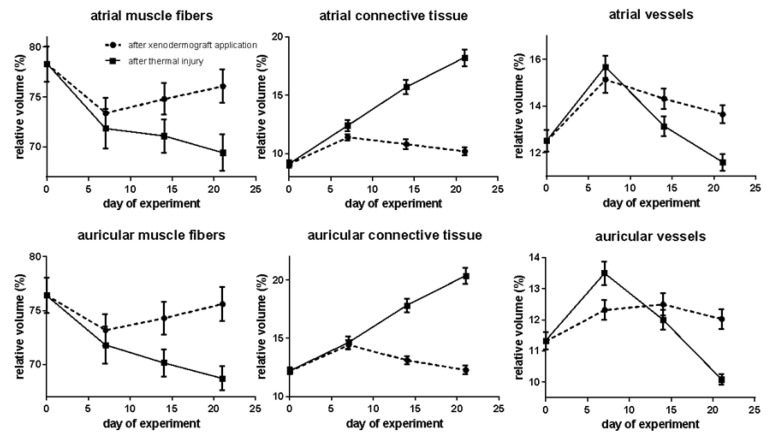
Relative volumes of the myocardium structures of the atria and auricles of the heart after thermal injury and in the condition of application of xenodermograft.

Distinct morphological features were found in the current investigation in the atrial myocardium due to the impaired blood supply to the heart. These features include the nuclear enlargement in the cytoplasm of cardiac muscle cells, as recently observed through electron microscopy. Our statistical analysis showed an increase in the percentage of loose connective tissue (1.25 times) and blood vessels (1.21 times) relative to the corresponding in dicators of intact animals. Compared to the animals in the second group, the relative volume of these structural components of the myocardium is 1.09 and 1.04 times lower, respec tively (Scheme 1).

In studies investigating the myocardial structure of burnt animals, changes were observed in the auricles of the heart, albeit less pronounced than those seen in the atria. Specifically, the relative volume of muscle fibers increased by 1.02 times relative to that of burnt animals but was 1.04 times lower than the normal level. The relative volume of connective tissue increased by 1.18 times relative to the rate of the intact animals and did not differ significantly from the corresponding rate of the burnt animals during this period. The percentage of myocardial vessels increased by 1.09 times compared to the normal range but decreased by 1.1 times compared to that of the burnt animals. When calculating the increased volume of hypertrophied cells (two-fold to three-fold), this reduction becomes even more significant (Scheme 1). These findings provide insights into the effects of impaired blood supply on the myocardial structure of auricles and may have implications for understanding cardiac function in the context of burn injury.

Exogenous and endogenous intoxication is a significant contributor to the changes observed in the myocardium following severe burns. Burn wounds are a common source of such intoxication, and consequently, reducing toxin levels in the body represents a promising avenue for treatment [8,9,10,11]. One area of recent interest in wound treatments is the development and application of biologically active dressings that can release substances that aid in reparative processes. Among these, lyophilized xenodermografts have seen increased usage in the field of combustiology for temporary wound surface closure [12]. Their application on cleansed wounds can prevent further intoxication from the wound, reduce the loss of essential nutrients from the surface, and promote faster skin regeneration. These benefits can have a positive impact on morphofunctional health [13,14,15,16].

Electron microscopy has revealed that the use of lyophilized xenodermotransplants in atrial cardiomyocytes has a beneficial impact on myocardial tissue. Specifically, regenerative processes were found to be dominant over destructive–degenerative ones, as evidenced by minimal damage to the cytoplasmic and nuclear membranes. Additionally, the nuclei were enlarged and predominantly composed of euchromatin, while ribosomal granules were present in the karyoplasm. Notably, the myofibrils maintained a normal structure, arranged in the order of sarcomeres. These findings indicate that the application of lyophilized xenodermografts for wound closure has a significant positive impact on the renewal of the intracellular structures of the heart.

The use of split-thickness lyophilized porcine skin has been demonstrated as an effective biological wound dressing that presents advantages in terms of convenience for storage, transport, and usage [13,28,41]. It serves as suitable temporary skin coverage for fresh burn wounds and wounds that remain after escharotomies, or as mesh grafts to prevent infections and the loss of body fluids, ultimately improving the general condition of the patient. Additionally, it can be applied to cover granulation wounds caused by burns or injuries to control bacterial growth and avoid further contamination. Nevertheless, lyophilized xenografts have a tendency towards autolysis and can only persist for a limited time, requiring repeated replacement to ensure a surgically clean wound surface suitable for autografts.

Developments in “humanizing” murine and porcine skin have promoted their potential efficacy in burn reconstruction in adults and pediatric patients [42]. Yet, there is no standardized procedure for xenograft use, resulting in a great variety of procedures with little scientific evidence.

Promising results have been seen with the use of xenografts in treating burn patients since the 1960s [43]. By covering denuded cutaneous surfaces, xenografts enhance granulation tissue and promote neo-vascularization as well as healing [44]. They are superior to other therapeutic options because large amounts of surface area can be covered (≤84 cm^2^). Because xenografts can cover an entire defect, they provide care-free wound management, rapid and easy application, and reduced pain at a low cost [44,45,46].

The present trend in research is towards use of pigs as donors. Transgenic pigs have been available for some years, and recent ‘knockout’ pigs have been generated by nuclear transfer techniques [47,48,49]. This means that we are now capable of removing or adding proteins to and from potential donor animals—a luxury clearly unavailable in allotransplantation. Additionally, pigs are bred for slaughter and their use should not generate the objections that arise with non-human primates. We already have extensive knowledge of husbandry conditions, and studies have shown the possibility of producing pigs with little or no infectious diseases [46,50]. Because of their phylogenetic distance from man, the likelihood of the cross-species transmission of infections is lower.

Over 100 cases of burns or injuries have been treated with this recommended method, revealing that lyophilized porcine skin is a satisfactory substitute for allografts, which frequently function as biological dressings [19,47,51,52].

## 5. Conclusions

The findings of this research demonstrate that severe thermal trauma resulting in deep and large skin burns induces notable morphofunctional changes in both the atria and auricles of the heart. These changes are directly linked to the stage of burn disease, which is primarily determined by the microcirculatory tract, cardiomyocytes, and vascular tissue ratios within the myocardium.

The application of lyophilized xenodermografts has been shown to have a positive effect on regenerative processes and can improve the structural components and morphometric parameters of the heart over time. These results suggest that further studies should be conducted to investigate the efficacy of corrective factors on the cardiovascular system in cases of severe thermal injury.

In summary, this study sheds light on the potential benefits of lyophilized xenodermografts as a promising approach for the treatment of severe burn wounds and their associated cardiovascular complications.

## Figures and Tables

**Table 1:** Relative volumes of the myocardium structures of the atria and auricles of the heart after thermal injury without using lyophilized xenodermotransplants.

Term of the Experiment	Muscle Fibers	Connective Tissue	Vessels
**Atria**			
Intact	78.32 ± 1.78	9.16 ± 0.32	12.52 ± 0.47
7th day	71.87 ± 1.97 *	1.44 ± 0.49 *	15.69 ± 0.47 *
14th day	71.13 ± 1.68 *	15.72 ± 0.62 *	13.15 ± 0.42
21st day	69.46 ± 1.82 *	18.24 ± 0.73 *	11.6 ± 0.36
**Auricles of heart**			
Intact	76.43 ± 1.63	12.24 ± 0.47	11.33 ± 0.28
7th day	71.82 ± 1.72 *	14.67 ± 0.53 *	13.51 ± 0.38 *
14th day	70.17 ± 1.26 *	17.82 ± 0.58 *	12.01 ± 0.32
21st day	68.73 ± 1.11 *	20.37 ± 0.71 *	10.09 ± 0.17 *

Note: * average values that significantly (*p* < 0.05) differ from those of intact animals.

**Table 2:** Relative volumes of the myocardium structures of the atria and auricles of the hearts of guinea pigs with severe burns receiving xenodermografts.

Term of the Experiment	Relative Volume (M ± m) %		
	Muscle Fibers	Connective Tissue	Vessels
**Atria**			
Intact	78.32 ± 1.78	9.16 ± 0.32	12.52 ± 0.47
7th day	73.42 ± 1.54 *	11.43 ± 0.27 *	15.15 ± 0.57 *
14th day	74.83 ± 1.58 *	10.84 ± 0.42 *	14.33 ± 0.43 *
21st day	76.11 ± 1.67	10.23 ± 0.36	13.66 ± 0.39
**Auricles of heart**			
Intact	76.43 ± 1.63	12.24 ± 0.27	11.33 ± 0.28
7th day	73.21 ± 1.47 *	14.46 ± 0.41 *	12.33 ± 0.32 *
14th day	74.33 ± 1.52 *	13.16 ± 0.34 *	12.51 ± 0.36 *
21st day	75.64 ± 1.58	12.32 ± 0.35	12.04 ± 0.32

Note: * average values that significantly (*p* < 0.05) differ from those of intact animals.

## Data Availability

Not applicable.

## References

[1] Farina J.A., Rosique M.J., Rosique R.G. (2013). Curbing Inflammation in Burn Patients. Int. J. Inflamm..

[2] Netiukhailo L., Kharchenko S., Kostenko A. (2010). Pathogenesis of burn disease (in 2 parts). World Med. Biol..

[3] Zhao Y., Feric N.T., Thavandiran N., Nunes S.S., Radisic M. (2014). The Role of Tissue Engineering and Biomaterials in Cardiac Regenerative Medicine. Can. J. Cardiol..

[4] Rudenko A.V., Vitovs’kyi R.M., Isaienko V.V., Krykunov O.A., Beshliaha V.M., Salo S.V., Fedoniuk L., Kryven’kyi O.V. (2007). Abscess of the mitral valve stimulating a tumor of the left ventricle. Klinichna Khiruriia.

[5] Nahaichuk V. (2010). Modern approaches to providing care to patients with burns. Art Treat..

[6] Murphy J.T., Purdue G.F., Hunt J.L. (1995). Pediatric Grease Burn Injury. Arch. Surg..

[7] Hart D., Wolf S., Mlcak R., Chinkes D., Ramzy P., Obeng M., Ferrando A., Wolfe R., Herndon D. (2000). Persistence of muscle catabolism after severe burn. Surgery.

[8] Jeschke M.G., Pinto R., Kraft R., Nathens A.B., Finnerty C.C., Gamelli R.L., Gibran N.S., Klein M.B., Arnoldo B.D., Tompkins R.G. (2015). Morbidity and Survival Probability in Burn Patients in Modern Burn Care. Crit. Care Med..

[9] Wang F., Guan J. (2010). Cellular cardiomyoplasty and cardiac tissue engineering for myocardial therapy. Adv. Drug Deliv. Rev..

[10] Chen Q.-Z., Harding S.E., Ali N.N., Lyon A.R., Boccaccini A.R. (2008). Biomaterials in cardiac tissue engineering: Ten years of research survey. Mater. Sci. Eng. R Rep..

[11] Mykhailovska N.S., Stetsiuk I.O., Kulynych T.O., Fedoniuk L.Y. (2020). The diagnostic and prognostic value of biomarkers in women with coronary artery disease and osteoporosis. Arch. Balk. Med. Union.

[12] Iakovlieva L.V., Koshova O.Y., Tkachova O.V., Gordienko A.D. (2018). Immunomodulatory properties of the cryopreserved pig skin substrate in an experiment. Clin. Pharm..

[13] Bihuniak V.V., Povstyanyi M.Y., Volkov K.S., Taran V.M., Nagaychuk V.I., Savchin V.S., Kiruk O.V., Guda N.V. (2003). The Use of Lyophilized Xenodermografts in Combustiology. Guidelines. Ternopil.

[14] Janak J.C., Clemens M.S., Howard J.T., Le T.D., Cancio L.C., Chung K.K., Gurney J.M., Sosnov J.A., Stewart I.J. (2018). Using the injury severity score to adjust for comorbid trauma may be double counting burns: Implications for burn research. Burns.

[15] Kallinen O., Maisniemi K., Böhling T., Tukiainen E., Koljonen V. (2012). Multiple Organ Failure as a Cause of Death in Patients with Severe Burns. J. Burn. Care Res..

[16] Kearney L., Francis E.C., Clover A.J. (2018). New technologies in global burn care—A review of recent advances. Int. J. Burn. Trauma.

[17] Bihuniak V.V., Povstianyi M.I. (2004). Termichni Urazhennia.

[18] Fedoniuk L.Y., Kulyanda I.S., Dovgalyuk A.I., Lomakina Y.V., Kramar S.B., Kulianda O.O., Valko O.O. (2021). Morphological characteristics of acellular dermal matrix manufacturing. Wiad. Lek..

[19] Hetmaniuk I., Fedoniuk L., Zhulkevych I., Andreychyn S., Hudyma A., Hanberher I., Dobrianskyi T. (2020). Microscopic and sub microscopic structure of the heart atria and auricles in condition of the experimental thermal trauma. Biointerface Res. Appl. Chem..

[20] Shin S.Y., Rios H.F., Giannobile W.V., Oh T.-J., Vishwakarma A., Sharpe P., Shi S., Ramalingam M. (2015). Chapter 36–periodontal regeneration: Current therapies. Stem Cell Biology and Tissue Engineering in Dental Sciences.

[21] Qu J., Zhao X., Liang Y., Zhang T., Ma P.X., Guo B. (2018). Antibacterial adhesive injectable hydrogels with rapid self-healing, extensibility and compressibility as wound dressing for joints skin wound healing. Biomaterials.

[22] Paggiaro A.O., Bastianelli R., Carvalho V., Isaac C., Gemperli R. (2019). Is allograft skin, the gold-standard for burn skin substitute? A systematic literature review and meta-analysis. J. Plast. Reconstr. Aesthetic Surg..

[23] Kitala D., Klama-Baryła A., Łabuś W., Kraut M., Glik J., Kawecki M., Joszko K., Gzik-Zroska B. (2020). Porcine Transgenic, Acellular Material as an Alternative for Human Skin. Transplant. Proc..

[24] Stoll J.R., Noor S.J., Dusza S.W., Markova A. (2021). Skin substitutes for the treatment of chronic wounds in cancer patients: A retrospective case series. J. Am. Acad. Dermatol..

[25] Fedoniuk L., Malyk Y. (2015). Typical tendon strings of the mitral valve and abnormally located strings of the left ventricle of the human heart. World Med. Biol..

[26] Fedoniuk L., Penteleichuk N. (2015). Morphological characteristics of tendon strings of the atrioventricular valves of the heart of fetuses, newborns and infants in norm. World Med. Biol..

[27] Chaudhuri R., Ramachandran M., Moharil P., Harumalani M., Jaiswal A.K. (2017). Biomaterials and cells for cardiac tissue engineering: Current choices. Mater. Sci. Eng. C.

[28] Weinberger F., Mannhardt I., Eschenhagen T. (2017). Engineering Cardiac Muscle Tissue. Circ. Res..

[29] Baar K., Birla R., Boluyt M.O., Borschel G.H., Arruda E.M., Dennis R.G. (2005). Self-organization of rat cardiac cells into contractile. 3-D cardiac tissue. FASEB J..

[30] Lemoine M.D., Mannhardt I., Breckwoldt K., Prondzynski M., Flenner F., Ulmer B., Hirt M.N., Neuber C., Horváth A., Kloth B. (2017). Human iPSC-derived cardiomyocytes cultured in 3D engineered heart tissue show physiological upstroke velocity and sodium current density. Sci. Rep..

[31] Sigdel K.P., Pittman A.E., Matin T.R., King G.M. (2018). High-Resolution AFM-Based Force Spectroscopy. Nanoscale Imaging Methods Protoc..

[32] Majid Q.A., Fricker A.T.R., Gregory D.A., Davidenko N., Cruz O.H., Jabbour R.J., Owen T.J., Basnett P., Lukasiewicz B., Stevens M. (2020). Natural Biomaterials for Cardiac Tissue Engineering: A Highly Biocompatible Solution. Front. Cardiovasc. Med..

[33] Copper J.E., Budgeon L.R., Foutz C.A., van Rossum D., Vanselow D., Hubley M.J., Clark D.P., Mandrell D.T., Cheng K.C. (2017). Comparative analysis of fixation and embedding techniques for optimized histological preparation of zebrafish. Comp. Biochem. Physiol. Part C: Toxicol. Pharmacol..

[34] Balan I., Khayo T., Sultanova S., Lomakina Y. (2021). Overview of Sodium-Glucose Co-transporter 2 (SGLT2) Inhibitors for the Treatment of Non-diabetic Heart Failure Patients. Cureus.

[35] Hogenhuis J., Voors A.A., Jaarsma T., Hillege H.L., Boomsma F., van Veldhuisen D.J. (2005). Influence of age on natriuretic peptides in patients with chronic heart failure: A comparison between ANP/NT-ANP and BNP/NT-proBNP. Eur. J. Heart Fail..

[36] Nishikimi T., Hagaman J.R., Takahashi N., Kim H.-S., Matsuoka H., Smithies O., Maeda N. (2005). Increased susceptibility to heart failure in response to volume overload in mice lacking natriuretic peptide receptor-A gene. Cardiovasc. Res..

[37] Kisch B. (1956). Electron microscopy of the atrium of the heart. I. Guinea pig. Exp. Med. Surg..

[38] Jamieson J.D., Palade G.E. (1964). Specific Granules in Atrial Muscle Cells. J. Cell Biol..

[39] De Bold A.J. (1986). Atrial natriuretic factor: An overview. Fed. Proc..

[40] Miller E.D. (1990). The Cardiac Patient and Atrial Natriuretic Hormone. Anesthesiology and the Heart.

[41] Hnatiuk M.S., Belikova N.O., Pryshliak A.M. (2001). Vikovi osoblyvosti sekretornoi aktyvnosti kardiomiotsytiv peredserd u ek-sperymentalnykh tvaryn. Nauk. Visnyk Uzhhorodskoho Universytetu Seriia “Medytsyna”.

[42] Zhurakivska O.I. (2003). Ultrastrukturnyi stan mioendokrynnykh klityn sertsia v normi. Halytskyi Likarskyi Visnyk.

[43] Hein S., Scholz D., Fujitani N., Rennollet H., Brand T., Friedl A., Schaper J. (1994). Altered Expression of Titin and Contractile Proteins in Failing Human Myocardium. J. Mol. Cell. Cardiol..

[44] Person V., Kostin S., Suzuki K., Labeit S., Schaper J. (2000). Antisense oligonucleotide experiments elucidate the essential role of titin in sarcomerogenesis in adult rat cardiomyocytes in long-term culture. J. Cell Sci..

[45] Davis D.A., Arpey C.J. (2000). Porcine Heterografts in Dermatologic Surgery and Reconstruction. Dermatol. Surg..

[46] Yang Y.W., Ochoa S.A. (2016). Use of Porcine Xenografts in Dermatology Surgery: The Mayo Clinic Experience. Dermatol. Surg..

[47] Burkey B., Davis W., Glat P.M. (2016). Porcine xenograft treatment of superficial partial-thickness burns in paediatric patients. J. Wound Care.

[48] Frankish H. (2002). Pig organ transplantation brought one step closer. Lancet.

[49] Weiss R., Magre S., Takeuchi Y. (2000). Infection Hazards of Xenotransplantation. J. Infect..

[50] Schaper J., Kostin S., Hein S., Elsässer A., Arnon E., Zimmermann R. (2002). Structural remodelling in heart failure. Exp. Clin. Cardiol..

[51] Gu B. (1990). Clinical use of lyophilized porcine skin. Zhonghua Zheng Xing Shao Shang Wai Ke Za Zhi Chin. J. Plast. Surg. Burns.

[52] Nguyen T.T., Gilpin D.A., Meyer N.A., Herndon D.N. (1996). Current Treatment of Severely Burned Patients. Ann. Surg..

